# Dexibuprofen ameliorates peripheral and central risk factors associated with Alzheimer’s disease in metabolically stressed APPswe/PS1dE9 mice

**DOI:** 10.1186/s13578-021-00646-w

**Published:** 2021-07-22

**Authors:** Miren Ettcheto, Elena Sánchez-Lopez, Amanda Cano, Marina Carrasco, Katherine Herrera, Patricia R. Manzine, Triana Espinosa-Jimenez, Oriol Busquets, Ester Verdaguer, Jordi Olloquequi, Carme Auladell, Jaume Folch, Antoni Camins

**Affiliations:** 1 Department of Pharmacology, Toxicology and Therapeutic Chemistry, Faculty of Pharmacy and Food Science, University of Barcelona, Barcelona, Spain; 2 Biomedical Research Networking Centre in Neurodegenerative Diseases (CIBERNED), Madrid, Spain; 3 Institute of Neuroscience, University of Barcelona, Barcelona, Spain; 4 Institute of Nanoscience and Nanotechnology (IN2UB), University of Barcelona, Barcelona, Spain; 5 Department of Pharmacy, Pharmaceutical Technology and Physical Chemistry, Faculty of Pharmacy and Food Science, University of Barcelona, Barcelona, Spain; 6 Research Center and Memory Clinic, Fundació ACE. Institut Català de Neurociències Aplicades – International University of Catalunya (UIC), Barcelona, Spain; 7 Department of Biochemistry and Biotechnology, Faculty of Medicine and Life Science, University Rovira I Virgili, Reus, Spain; 8 Department of Cellular Biology, Physiology and Immunology, Faculty of Biology, University of Barcelona, Barcelona, Spain; 9 Department of Gerontology, Federal University of São Carlos (UFSCar), São Carlos, 13565-905 Brazil; 10 Dominick P. Purpura Department of Neurosciences, Albert Einstein College of Medicine, New York City (10461), USA; 11 Laboratory of Cellular and Molecular Pathology, Facultad de Ciencias de La Salud, Instituto de Ciencias Biomédicas, Universidad Autónoma de Chile, Talca, Chile; 12 Unitat de Farmacologia I Farmacognòsia, Facultat de Farmàcia I Ciències de L’Alimentació, Universitat de Barcelona, Av. Joan XXIII 27/31, 08028 Barcelona, Spain

**Keywords:** APPswe/PS1dE9, Alzheimer´s disease, Dexibuprofen, Unfolded protein response, neuroinflammation, Synapsis, Cognitive deficits, High fat diet, Metabolic alterations, βA plaques

## Abstract

**Background:**

Several studies stablished a relationship between metabolic disturbances and Alzheimer´s disease (AD) where inflammation plays a pivotal role. However, mechanisms involved still remain unclear. In the present study, we aimed to evaluate central and peripheral effects of dexibuprofen (DXI) in the progression of AD in APPswe/PS1dE9 (APP/PS1) female mice, a familial AD model, fed with high fat diet (HFD). Animals were fed either with conventional chow or with HFD, from their weaning until their sacrifice, at 6 months. Moreover, mice were divided into subgroups to which were administered drinking water or water supplemented with DXI (20 mg kg^−1^ d^−1^) for 3 months. Before sacrifice, body weight, intraperitoneal glucose and insulin tolerance test (IP-ITT) were performed to evaluate peripheral parameters and also behavioral tests to determine cognitive decline. Moreover, molecular studies such as Western blot and RT-PCR were carried out in liver to confirm metabolic effects and in hippocampus to analyze several pathways considered hallmarks in AD.

**Results:**

Our studies demonstrate that DXI improved metabolic alterations observed in transgenic animals fed with HFD in vivo, data in accordance with those obtained at molecular level. Moreover, an improvement of cognitive decline and neuroinflammation among other alterations associated with AD were observed such as beta-amyloid plaque accumulation and unfolded protein response.

**Conclusions:**

Collectively, evidence suggest that chronic administration of DXI prevents the progression of AD through the regulation of inflammation which contribute to improve hallmarks of this pathology. Thus, this compound could constitute a novel therapeutic approach in the treatment of AD in a combined therapy.

**Supplementary Information:**

The online version contains supplementary material available at 10.1186/s13578-021-00646-w.

## Background

Alzheimer´s disease (AD) is a progressive neurodegenerative disease characterized by severe impairments of cognitive function leading to development of drastic dementia. For years, this pathology has been defined by the combined presence of amyloid-β (Aβ) plaques and neurofibrillary tangles composed by hyperphosphorylated Tau. However, the continuous increase of cases worldwide together with the failure of proposed treatments until now have located this pathology as one of the great health-care challenges of the twenty-first century, promoting new approach about its etiology and, therefore, about its therapeutic strategies.

In this regard, in the recent years, several studies have focused on the potential relationship between AD and metabolic disorders [1, 2], considering AD as a multifactorial disease. In fact, longitudinal studies have indicated worsening performance of specific abilities including working memory in patients with metabolic disorders [3, 4]. In line with this hypothesis, obesity and type 2 diabetes mellitus (T2DM) also have been associated to cognitive decline in several studies considering them as key contributors not only in cognitive alterations [5–8] but also in the development of AD [9, 10]. As a result, a new concept have been introduced by scientific community to define AD named “type 3 diabetes” [11, 12].

In this context, the association between T2DM and AD is very complex where several molecular pathways are interlinked such as insulin resistance, peripheral inflammatory response, and endoplasmic reticulum stress, among others [13]. Likewise, insulin resistance is one of the main hallmarks of obesity and T2DM which plays an essential role in the maintenance in the cell energy homeostasis. Surprisingly, the appearance of similar abnormalities in both, peripheral tissues of T2DM patients and AD brains has been demonstrated [14–17]. Moreover, Wu and coworkers have proven the existence of peripheral metabolic changes in plasma and liver of AD mice models, suggesting that AD development is not only caused by alterations in the brain but also that systemic impairment plays a key role in the pathology [18]. In fact, it has also been demonstrated that peripheral alterations in the insulin pathway observed in patients with T2DM contribute to alterations in brain insulin, leading to an increase of Aβ accumulation and a decrease of its clearance, which induce neuronal damage and, therefore, cognitive decline, creating a vicious cycle of pathogenesis [19].

As it has been mentioned before, inflammation is involved not only metabolic disorders but also in AD, and is considered a critical process in this pathology. However, its dual contribution to the disease adds complexity to the process. Moreover, increasing clinical and experimental studies have evidenced that acute and chronic systemic inflammatory pathologies may be associated with the risk and acceleration of AD [20]. In line with this hypothesis, numerous epidemiological studies have demonstrated that anti-inflammatory drugs can reduce the risk of development AD in more than 50% fold [21–23], for this reason non-steroidal anti-inflammatory drugs are being one the research focus concerning to AD therapeutic strategy.

Ibuprofen (IBU) is one of the most used nonsteroidal anti-inflammatory drugs (NSAIDs) which has demonstrated promising results in decreasing hallmarks of AD [24], by contrast, its chronic intake has demonstrated multiple adverse effect such as gastric damage. For these reasons, our study has focused in the evaluation of dexibuprofen (DXI), the active enantiomer of IBU [25, 26], which has been demonstrated that it has not only a more powerful anti-inflammatory and analgesic effect compared to IBU but also less ulcerogenic side effects [27–30]. Therefore, taking these previous findings into account, and according to the Herrup hypothesis which suggests that peripheral inflammation could be one of the responsible of neuroinflammation associated to neurodegeneration, the aim of our study is to investigate the central and peripheral effects of DXI in the progression of AD and describe the involved mechanisms in APPswe/PS1dE9 (APP/PS1) female mice, familial AD mice model, fed with high fat diet (HFD).

## Methods

### Animals and treatment

In this study, six-month old female APP/PS1 double transgenic mice were used. Female sex was chosen due to the fact that it has been reported that they produce a significantly higher amyloid burden and subsequent plaque deposition than their male counterparts [31]. Moreover, these transgenic mice express a Swedish (K594M/N595L) mutation of a chimeric mouse/human APP gene (mo/huAPP695swe) together with the human exon-9-deleted variant of presenilin 1 (PS1-dE9). Animals were divided into 4 groups: (1) mice fed with conventional chow (APP/PS1), (2) mice fed with conventional chow and additionally treated with DXI (APP/PS1 DXI), (3) mice fed with a palmitic acid-enriched high fat diet (APP/PS1 HFD) containing 45% of fat mainly from hydrogenated coconut oil (Research Diets Inc, New Brunswick, USA) (APP/PS1 HFD) and (4) mice fed with HFD and additionally treated with DXI (APP/PS1 HFD DXI). The drug was administered in drinking water at a dose of 20 mg kg^−1^ d^−1^ for 3 months, from mice aged 3 months old until their sacrifice at 6 months, as previously described [30]. At least, 10 animals per group were used for this study. All mice were given access to water and food ad libitum and kept under controlled light, temperature and humidity conditions. Every possible effort was made to reduce the number of animals used and minimize their suffering. Mice were treated in accordance with the European Community Council Directive 86/609/EEC and the procedures were established by the Department d’Agricultura, Ramaderia i Pesca of the Generalitat de Catalunya.

### Glucose and insulin tolerance test

Intraperitoneal glucose tolerance test (IP-GTT) and insulin tolerance test (IP-ITT) were performed in accordance with the previously described guideline [32]. Briefly, mice were fasted, at least, for 6 h prior to carry out both procedures and at least 10 animals were used per group. Tail blood was collected at multiple time points. Blood glucose levels were assayed with Accu-Chek Aviva glucometer (Roche; Mannheim, Germany). Those animals in which blood glucose concentration reached values below 20 mg/dl were treated with glucose at a dose of 1 g/kg.

### Cognitive test

#### Morris water maze

APP/PS1 mice were subjected to Morris water maze (MWM) test in order to assess the spatial memory and learning abilities as previously described by our group [33]. Acquired data were analyzed using SMART V3.0 (Panlab Harvard Apparatus, Germany) video tracking system, calculating results individually for each animal. At least, 10 animals per group were utilized.

#### Novel object recognition test

The novel object recognition test (NORT) was used for assessing the hippocampal-dependent recognition memory of mice as it has been previously detailed [34]. At least, 10 mice per condition were used. Data were analyzed by discrimination index (DI) which was calculated using the following equation:

$$DI= \frac{New\, object \,exploration \,time-old \,object \,exploration \,time}{total \,exploration \,time}$$

Data measured and represented in seconds.

### Immunofluorescence, S-thioflavin staining and enzymatic immunohistochemistry

To perform these 3 techniques, 4–5 mice were anesthetized by i.p. injection of ketamine (100 mg/kg) and xylacine (10 mg/kg) and perfused with 4% paraformaldehyde (PFA) diluted in 0.1 M phosphate buffer (PB). Brains were removed and stored in the same solution overnight (O/N). 24 h later, they were cryoprotected in 30% sucrose-PFA-PB solution for at least 1 day and samples were kept at − 80 °C until their use. Coronal sections of 20 μm of thickness were obtained using a cryostat (Leica Microsystems, Wetzlar, Germany).

To perform the experiments, free-floating technique was used for all of them. Immunoshistochemistry and S-thioflavin protocols were carried out as previously described [35]. Briefly, free-floating sections were rinsed in phosphate-buffered saline (PBS) (pH 7,2) prior to pre-incubation in a blocking solution (10% fetal bovine serum (FBS), 1% of triton X-100 in PBS at room temperature for 1 h and then, they were incubated O/N at 4 °C with the corresponding primary antibody and immediately with the corresponding secondary antibody for 2 h at room temperature.

Enzymatic immunohistochemistry was performed as previously described by our group [36]. All the antibodies used for these experiments have been described in Table 1.

**Table 1:** Primary and secondary antibodies for immunohistochemistry and immnunofluorescence

Protein	Reference
DBN1	ABN 207 (Merck Millipore)
GFAP	Z0334 (Dako)
IBA1	O19-19,741 (Wako)
Synaptophisin	MO776 (Dako)
2^nd^-ary Anti-rabbit IgG Biotin antibody produced in goat	B8895-1ML (Sigma Millipore)
2^nd^-ary AlexaFluor 488 (Goat anti-mouse)	A11001 (Life Technologies)
2^nd^-ary AlexaFluor 594 (Goat anti-rabbit)	A11080 (Life Technologies)

All the samples were mounted onto gelatinized slides with Fluoromount G (EMS). Image acquisition was performed with an epifluorescence microscope (BX41, Laboratory Microscope, NY-Olympus America Inc.). For plaque quantification, similar and comparable histological areas, particularly from the cortex, were selected. Immunohistochemistry labeling was analyzed through ImageJ software.

### Immunoblot analysis

For Immunoblot, 5–6 mice from each experimental group were sacrificed by cervical dislocation and their hippocampus and liver were dissected. Total protein extraction was carried out by homogenizing tissue samples in lysis buffer (50 mM Tris HCl pH: 7.4, 150 mM NaCl, 5 mM EDTA, 1%Triton X-100) containing a protease and phosphatase inhibitor mixture (Complete, Roche Diagnostics, Barcelona, Spain). Protein concentration was determined by Pierce BCA Protein Assay Kit (Pierce Company, Rockford, MI, USA) and aliquot of samples containing 10 μg were used for performing western blot procedure as previously described by our group [34]. The antibodies employed in the experiment are listed in Table 2. Finally, blots were exposed to Chemiluminescence-based detection kit in a Chemidoc™ XRS + Molecular Imager detection system (Bio-Rad), through Image Lab™ image analysis software (version 5.2.1). Obtained results were normalized to the corresponding glyceraldehyde 3-phosphate dehydrogenase (GAPDH) and expressed in arbitrary units (a.u).

**Table 2:** Primary and secondary antibodies for Western Blotting

Protein	Reference
ADAM10	ab39177 (Abcam)
AKT	#9272 (Cell Signaling Technology)
pAKT (S473)	#4060 (Cell Signaling Technology)
eIF2α	#9722 (Cell Signaling Technology)
peIF2α (S51)	#9721 (Cell Signaling Technology)
GAPDH	MAB374 (Millipore)
GSK3β	#9315 (Cell Signaling Technology)
pGSK3β (S9)	#9336 (Cell Signaling Technology)
IREα	Sc 390,960 (Santa Cruz Biotechnology)
pIREα (S724)	NB100-2323 (Novusbio)
IRS2	Ab134101 (Abcam)
pIRS2 (S731)	Ab3690 (Abcam)
2^nd^-ary Goat anti-rabbit	31,460 (Invitrogen)
2^nd^-ary Goat anti-mouse	31,430 (Invitrogen)

### Real time PCR

Samples were obtained as described in the *2.5 Immunoblot test* section where 4–6 animals were used. RNA extraction was performed by TRIsure-based extraction (Bloline GmbH) as previously described by our group [34]. The RNA pellet was reconstituted in RNAse free water and RNA concentration was measured using a NanoDropTM 1000 Spectrophotometer (Thermo Scientific, MA, USA). 2 μg of RNA was reversely transcribed with the High-Capacity cDNA Reverse Transcription Kit, according to manufacturer’s protocol (Applied Biosystems). To perform the real time PCR (RT-PCR), equal cDNA amounts of each animal were selected using as a reagent SYBR Green® qPCR Master Mix (K0253, Pierce, Thermo Fisher Scientific) in a Step One Plus™ Real-Time PCR System (Applied Biosystems). Samples were run by triplicate and data were normalized to *Gapdh*. Primers of detected genes are detailed in Table 3.

**Table 3:** Genes analyzed by RT-PCR

Gene	Forward	Reverse
*Atf3*	CTGGAGATGTCAGTCACCAAGTCT	TTTCTCGCCGCCTCCTTT
*Atf4*	AGCAAAACAAGACAGCAGCC	ACTCTCTTCTTCCCCCTTGC
*Atf6*	TTTCAGGGCAGGGCCATT	CCCGGGACAAACAGGTCTT
*Bip*	CAGATCTTCTCCACGGCTTC	GCAGGAGGAATTCCAGTCAG
*Chop*	CGAAGAGGAAGAATCAAAAACCTT	GCCCTGGCTCCTCTGTCA
*Gapdh*	TCTACCCACGGGCAAGTTCAA	GGTTTCTCCAGGCGGCATGT
*Ir*	TGTCCCCAGAAAAACCTCTTCA	AAGGGATCTTCGCTTTCGGG
*Irs1*	ACGAACACTTTGCCATTGCC	CCTTTGCCCGATTATGCAGC

### Statistical analysis

Differences between animals/samples were analyzed by two-way ANOVA, comparing two variables (treatment and diet). In this test, when the interaction between 2 factors was not significant, statistical values were represented through # where # p < 0.05, ## p < 0.01, ### p < 0.001 and #### p < 0.0001). When significant interaction was obtained, Tukey´s post-hoc test was performed to compare the different groups where significant values were represented by * (* p < 0.05. ** p < 0.01, *** p < 0.001 and **** p < 0.0001). All data are presented as mean ± SEM, in the Graph Pad 6.0 Prism software (Graph Pad Software Inc., San Diego, CA, USA).

## Results

### DXI treatment ameliorates HFD-related peripheral alterations in APP/PS1 mice

**Fig. 1:**
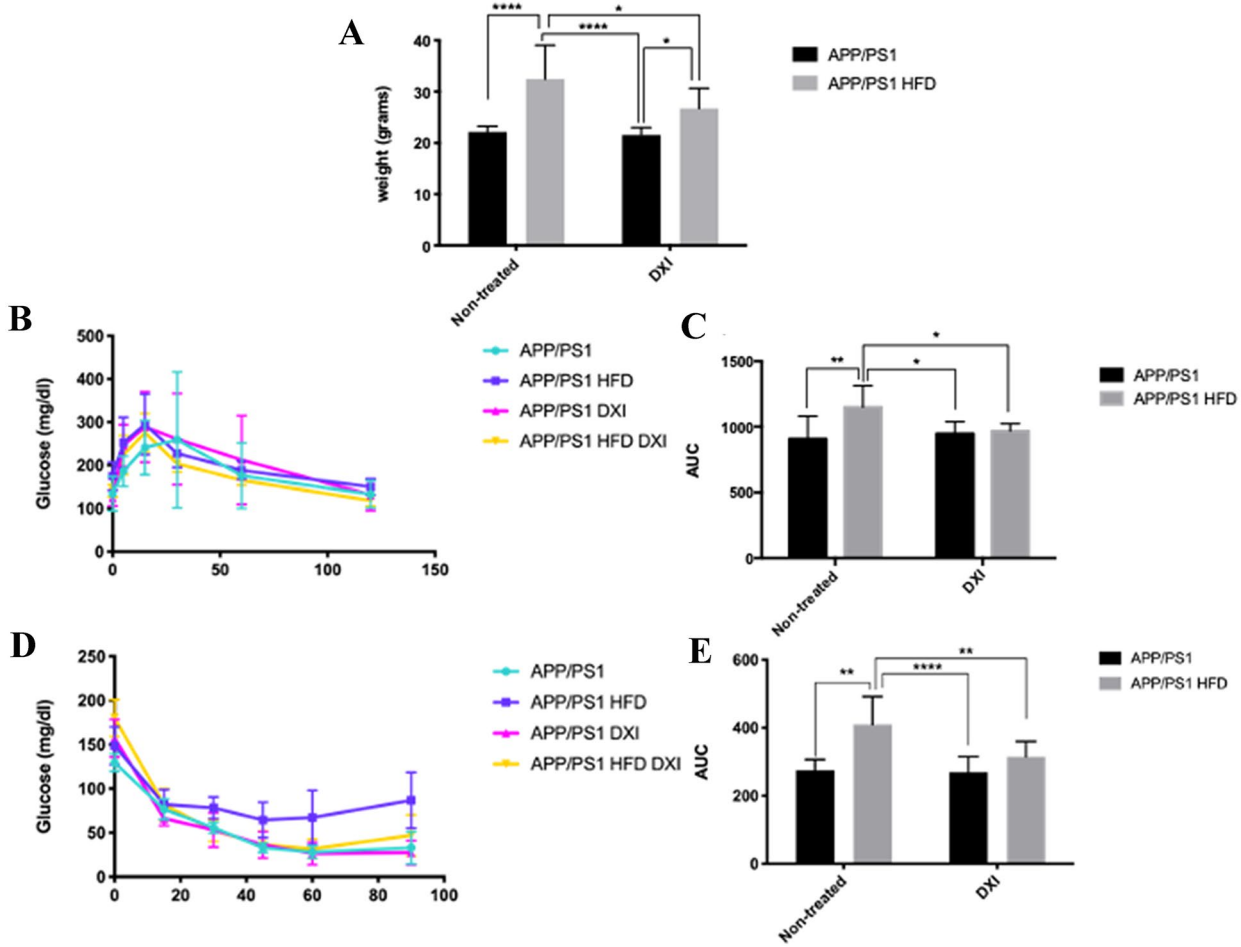
Body weight comparison of the different experimental groups (n = 8–10 mice per group) **B** IP-GTT and **D** IP-ITT experimental profiles (n = 8–10 independent samples per group). AUC was calculated from the timepoint 0 until the end of the experiments for both test **C** IP-GTT and **E** IP-ITT. Statistical analysis was performed through two-way ANOVA and Tukey´s post-hoc where * denotes p < 0.05; ** denotes p < 0.01 and **** denotes p < 0.0001

In order to evaluate the effect of DXI treatment on metabolic parameters after HFD intake in APP/PS1 mice, firstly, body weight measurement and IP-GTT and IP-ITT were performed and immediately protein level of molecules involved in insulin signaling pathway were evaluated. Two-way ANOVA of the weight gain revealed a significant effect of diet (p < 0.0001) and treatment (p < 0.05) with interaction between both variables (p < 0.05). As expected, animals fed with HFD showed a significant increase of body weight compared to those fed with conventional chow (p > 0.0001 for non-treated animals; p < 0.05 for treated animals). Surprisingly, mice fed with HFD and treated with DXI significantly reduced this parameter reaching values similar to animals fed with normal chow (p > 0.05) (Fig. 1A). Moreover, the weight gain observed in APP/PS1 HFD mice was accompanied by alterations in peripheral glucose metabolism in both, IP-GTT and IP-ITT. As previously described by our group and also by other authors [32, 37], two-way ANOVA showed a significant effect of diet in both test, IP-GTT and IP-ITT respectively (p < 0.05; p < 0.0001)), and in this last test also in the treatment factor (p < 0.05) with interaction between both factors in both test (p < 0.05; p < 0.05). Specifically, Tukey’s post-hoc revealed that HFD intake induced a significant increase of fasting glucose levels in response to glucose and insulin intraperitoneal injection in comparison to mice fed with conventional chow (p < 0.01; p < 0.01, respectively). By contrast, the results obtained in APP/PS1 HFD after DXI treatment were intriguing. While the glucose levels observed in APP/PS1 HFD group pointed out metabolic disturbances, animals fed with the same diet but with an additional DXI treatment showed a significant decrease of fasting glucose levels in both test, IP-GTT and IP-ITT, compared to their controls (p < 0.05; p < 0.01), achieving glucose concentrations similar to those animals fed with conventional chow (Fig. 1B–E).

**Fig. 2:**
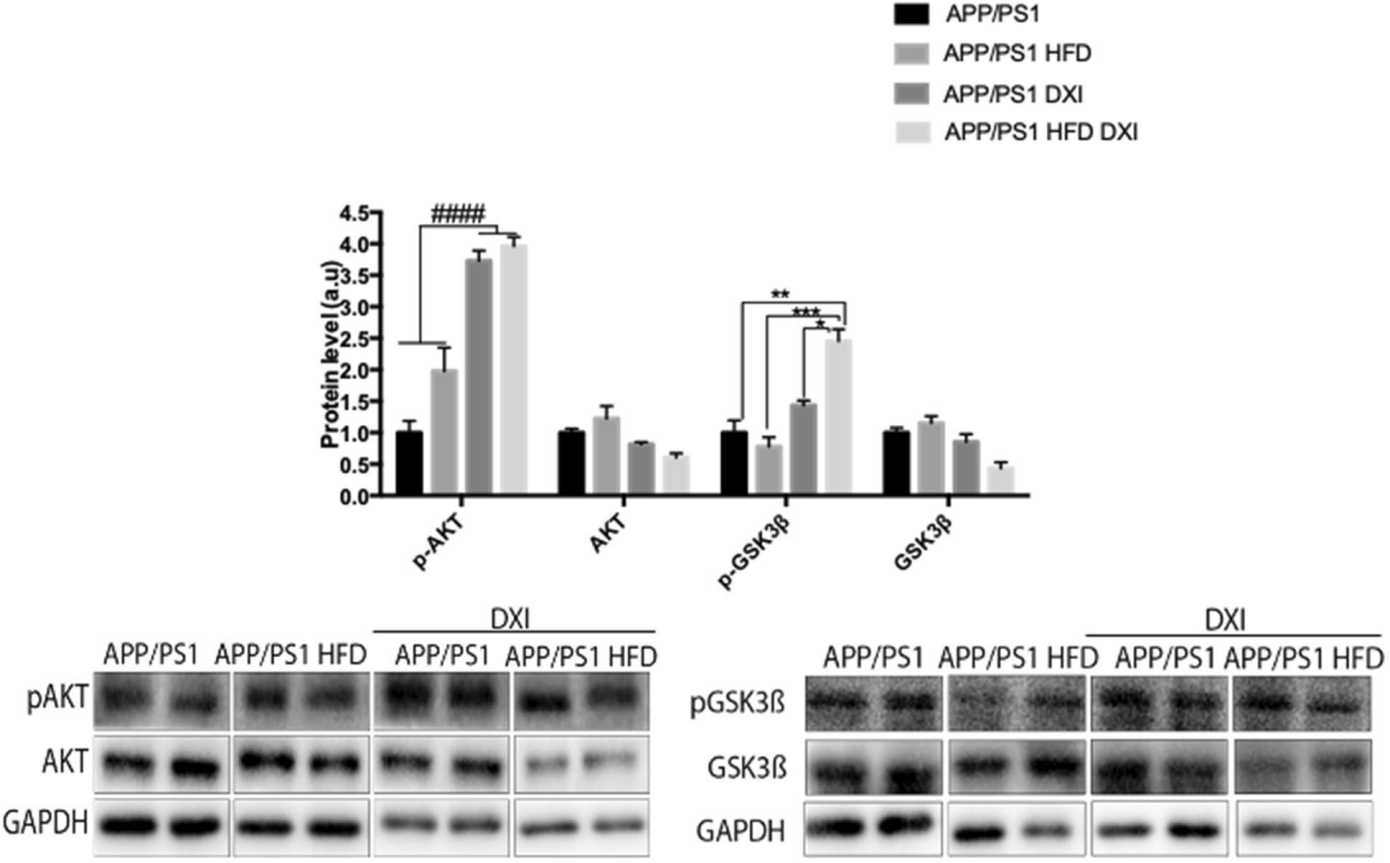
**A**–**C** Immunoblot representative images and GAPDH-normalized quantifications of molecules related to insulin signalling (pAKT/AKT and pGSK3β/GSK3β in liver extracts of APP/PS1, APP/PS1 HFD, APP/PS1 DXI and APP/PS1 HFD DXI experimental groups (n = 4–6 independent samples per group). Statistical analysis was performed through two-way ANOVA. Significant differences were found treatment variable: #### denote p < 0.0001

At molecular level, proteins involved in the insulin signaling pathway in liver were analyzed to confirm peripheral metabolic alterations. In line with observed previous data in this study, two-way ANOVA demonstrated a significant effect of treatment variable (p < 0.0001) in phospho protein kinase B (p-AKT) protein level. In the case of phospho glycogen synthase kinase 3 beta (p-GSK3β), our data also showed a significant effect of treatment (p < 0.001) with interaction between both variables (p < 0.01). Specifically, our data demonstrated a significant increase in APP/PS1 mice fed with HFD and treated to DXI group in comparison to non-treated group (p < 0.001). However, although upward trend was observed in animals fed with conventional chow after DXI treatment, no significant differences were observed. Lastly, AKT and GSK3β did no show differences among experimental groups (Fig. 2).

### DXI treatment improves cognitive decline observed in both APP/PS1 mice fed with conventional chow and obesogenic diet

**Fig. 3:**
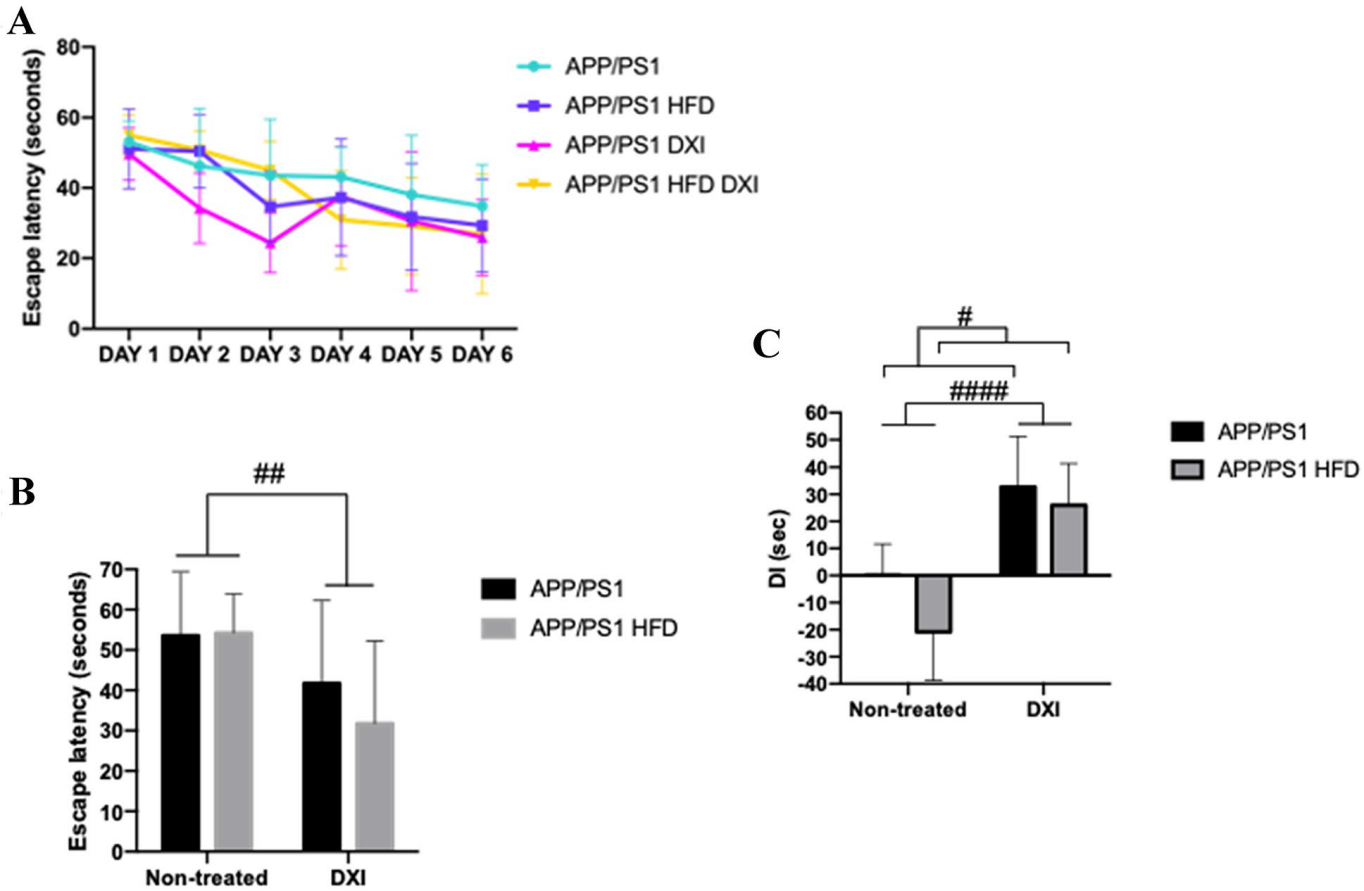
Behavioural test **A** Scape latency of training in MWM and **B** Scape latency of probe trial. All data were measured in seconds. Statistical analysis was performed through two-way ANOVA. Significant differences were found in treatment variable: ## denote p < 0.01 **C** NORT, DI expressed in seconds. (n = 10 independent samples per group. Two-way ANOVA statistical analysis was performed (Treatment variable: #### denote p < 0.0001) (Diet variable: # denote p < 0.05)

In previous studies, it has been demonstrated that APP/PS1 mice showed cognitive decline in terms of spatial learning and memory [30, 38, 39]. Therefore, we evaluated whether DXI was able to rescue the cognitive impairment observed in this mice model through both tests, MWM and NORT. Regarding MWM, as it is shown in the Fig. 3A, B, the training performed by different groups showed an improvement of learning ability in those animals treated with DXI compared to non-treated animals. In line with these results, 2-way ANOVA analysis showed a significant effect of the treatment variable in the test day (p < 0.01) independent of the diet that they had consumed (Fig. 3A, B). Moreover, results observed in the NORT after two-way ANOVA indicated a significant effect of both analyzed variables, treatment and diet (p < 0.0001; p < 0.05) respectively, demonstrating that DXI treatment improves cognitive decline previously described by our group not only in transgenic animals fed with conventional chow [30] but also in those fed with HFD (Fig. 3C). No significant differences were observed on the speed among any of the groups of the test day. Therefore, results of scape latency were not due to differences in the swimming speed of the animals. These results are attached as supplementary material.

**Fig. 4:**
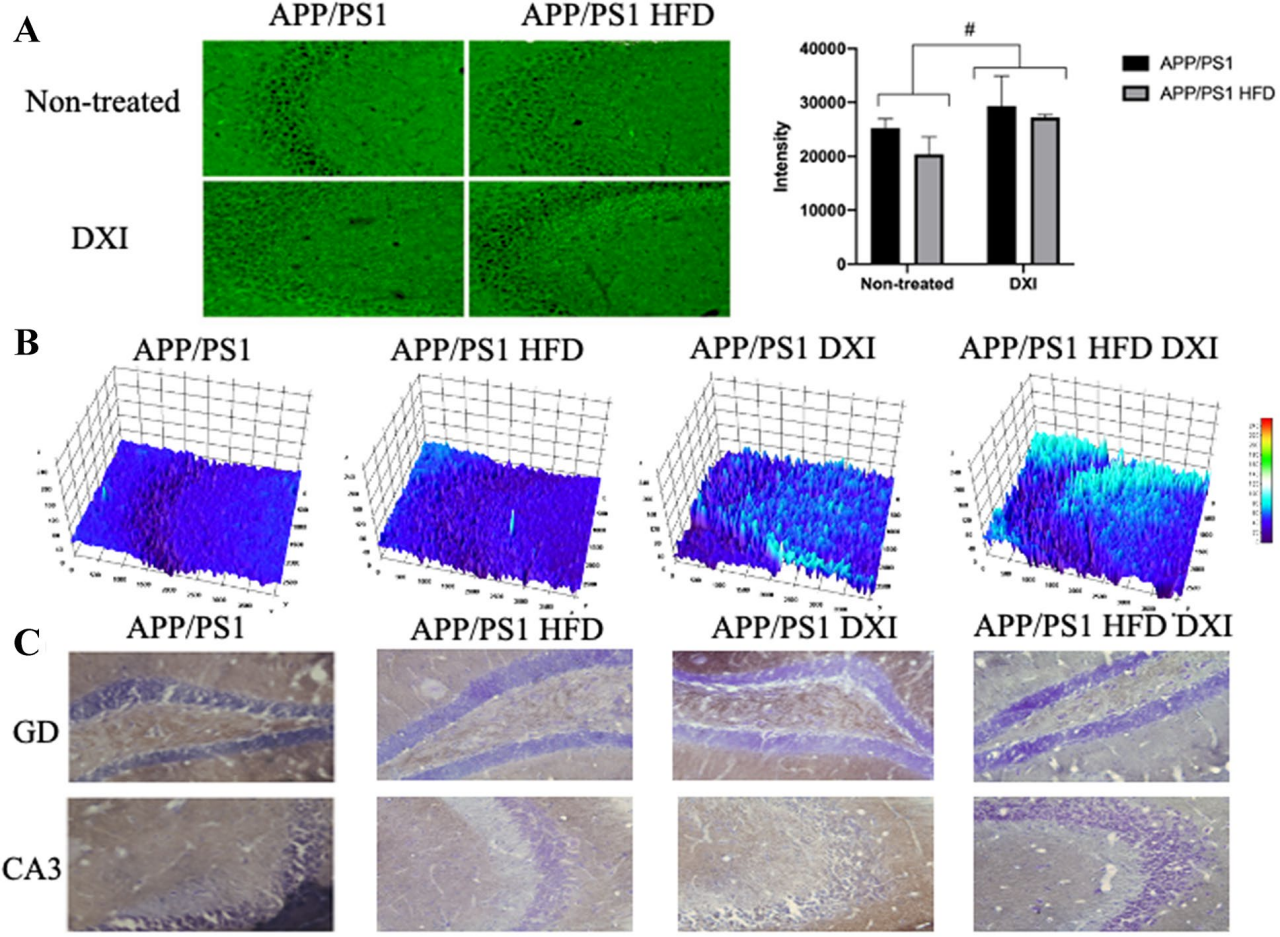
**A** Immunofluorescence against synaptophysin in the hippocampal CA3 areas and quantification of the intensity staining. Two-way ANOVA analysis was performed (Treatment variable: # denote p < 0.05). **B** 3D surface mapping analysis. Interactive 3D surface Plot v 2.4, ImageJ. Higher tridimensional relief and colour intensity were correlated with higher protein level. Scale 50 µm. **C** Immunofluorescence against DBN1 in the hippocampal GD and CA3 areas. Scale 50 µm

In order to support these results, an immunofluorescence assay against synaptophysin was carried out. In line with the results observed in the behavioral test, our results showed that the DXI treatment induced a substantial qualitative increase of this protein in the CA3 of the hippocampus compared to non-treated groups (Fig. 4A, B). In agreement with these results, the quantitative analysis of these images showed the significant effect of treatment variable (p < 0.005) after the application of two-way ANOVA. Moreover, these data were corroborated with an enzymatic immunohistochemistry against drebrin (DBN1). As it can be observed in the image, mice treated with DXI showed a clear increase in DBN1 protein level in CA3 and gyrus dentatus (GD) of the hippocampus (Fig. 4C).

### DXI reduces Aβ-plaque burden in APP/PS1 mice fed with conventional chow and obesogenic diet

**Fig. 5:**
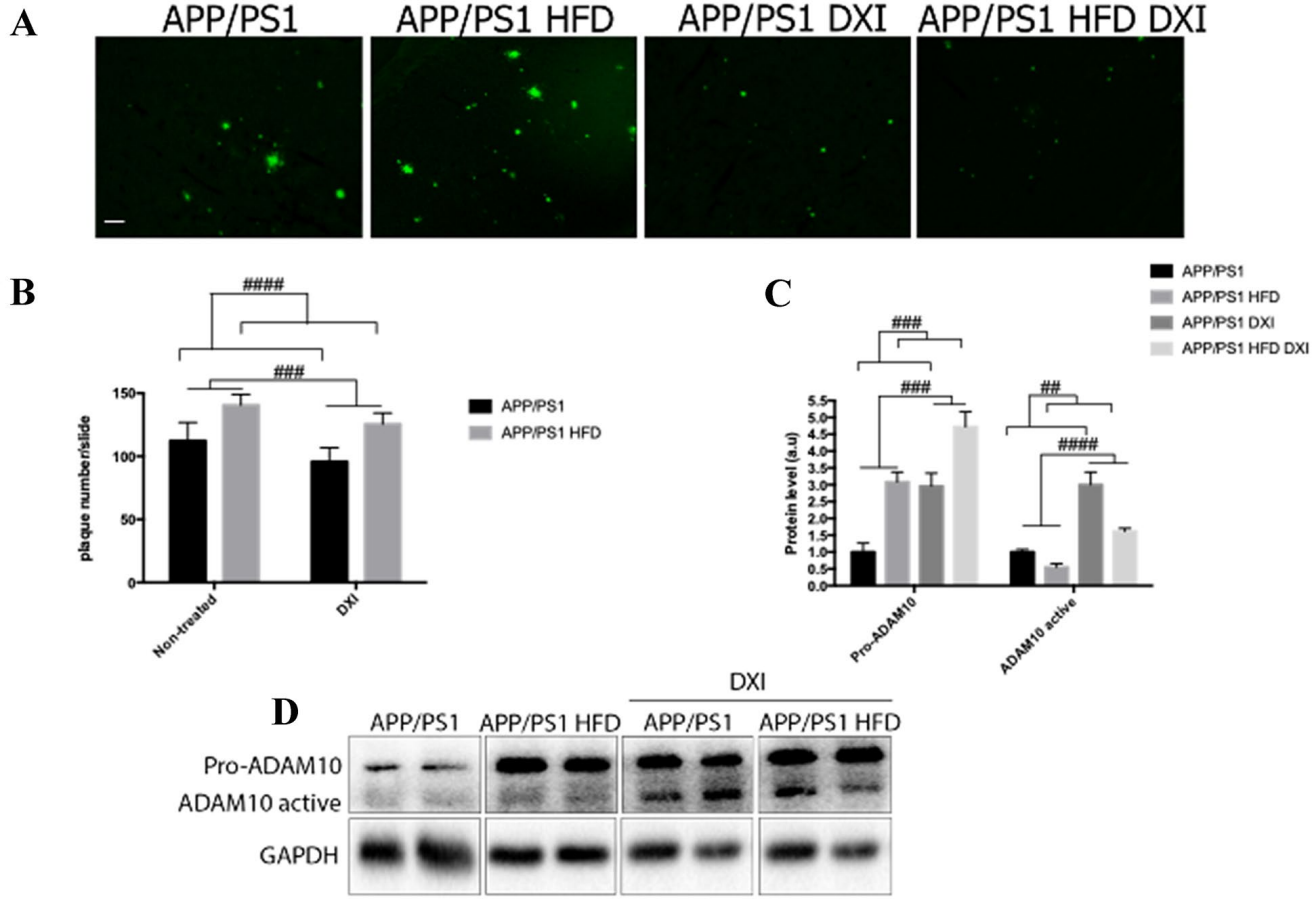
**A** Thioflavin staining of βA plaques (Green). Images were obtained from cortex. Scale bar 50 µm. **B** Representative histogram of the quantification of Aβ deposits (n = 4–6 independent samples per group, with at least 5 slices analyzed per simple). Two-way ANOVA statistical test was performed (Treatment variable: ### denote p < 0.001) (Diet variable: #### denote p < 0.0001). **C**, **D** Immunoblot representative images and GAPDH-normalized quantifications of molecules related to non-amyloidogenic pathway (pro-ADAM10 and ADAM10) from hippocampal extracts (n = 4–6 independent samples per group). Statistical analysis was performed through two-way ANOVA. (Pro-ADAM10, treatment and diet variables: ### denote p < 0.001). (ADAM10, treatment variable: ####denote p < 0.0001 and diet variable: ## denote p < 0.01)

It is widely demonstrated that APP/PS1 mice develop time-dependent accumulation of Aβ plaques in the brain, which is enhanced with the obesogenic diet intake [40, 41]. To evaluate the effect of DXI on this process, S-thioflavin staining was performed for plaque detection. As expected, two-way ANOVA showed a significant effect of the diet (p < 0.0001) in the quantification of cortical Aβ plaques deposition in APP/PS1 mice, demonstrating an increase of these plaques caused by HFD intake. Moreover, treatment factor also results in a significant effect (p < 0.0001), indicating that long-term administration of DXI reduced Aβ plaques deposition independent of the diet (Fig. 5A).

Likewise, Western blot analysis was carried out to evaluate the effect of DXI in non-amyloidogenic pathway, which is considered as the neuroprotective route [42]. In correlation with previously showed data, two-way ANOVA of the results of pro-disintegrin and metalloproteinase domain-containing protein 10 (ADAM10) and ADAM10 protein levels revealed a significant effect of diet (p < 0.001) and treatment (p < 0.001), confirming that both factors alter the protein levels of this protein (Fig. 5B–D).

### DXI treatment reduces the neuroinflammatory response in APP/PS1 fed with conventional chow and obesogenic diet

**Fig. 6:**
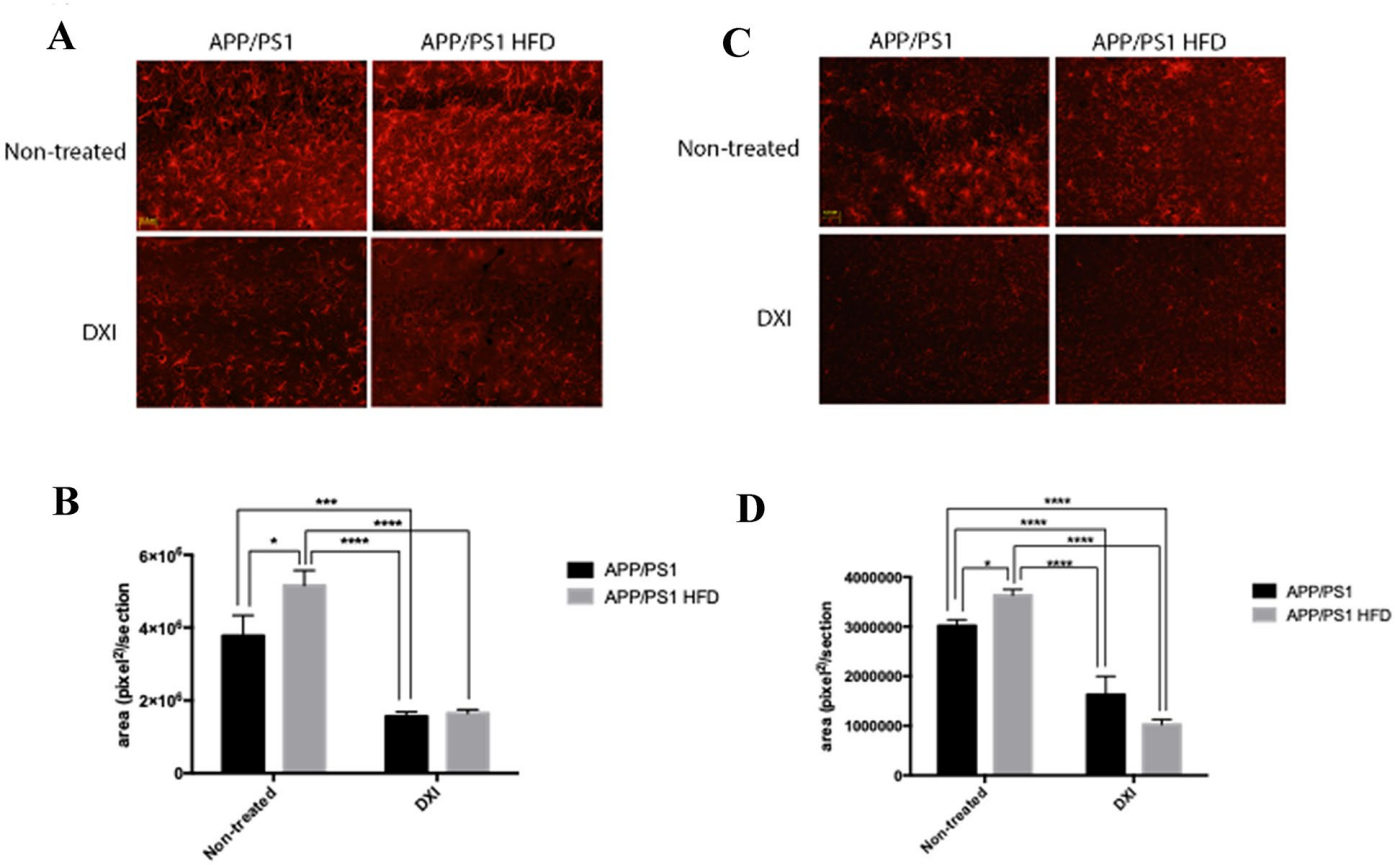
**A** Immunofluoresce against GFAP (astrocytes in red). **B** Representative histogram of quantification of astrocytes reactivity. **C** Immunofluoresce against Iba1 (microglia in red). **D** Representative histogram of quantification of microglial reactivity. Scale bar: 31,8 µm. Images were taken from the CA1 of hippocampus. (n = 4–6 independent samples per group, with at least 5 slices analyzed per sample). Statistical analysis was performed by two-way ANOVA and Tukey´s post-hoc where * denotes p < 0.05; *** denotes p < 0.001 and **** denotes p < 0.0001

It is well known that neuroinflammation plays a key role in the development of AD [43, 44], process which is exacerbated by the HFD intake [45–47]. In line with these previous data, two-way ANOVA of glial fibrillary acidic protein (GFAP) quantification indicated a significant implication of the treatment variable (p < 0.0001) and diet variable (p < 0.05) with interaction between both factors (p < 0.05). Moreover, two-way ANOVA for ionized calcium binding adaptor molecule 1 (IBA1) protein levels demonstrated a significant effect of treatment (p < 0.0001) with interaction between both variables (p < 0.01). Specifically, Tukey´s post-hoc demonstrated that HFD consumption show a higher reactive profile when they were quantified by their covered area in APP/PS1 fed with HFD compared to transgenic mice fed with conventional chow (p < 0,05; p < 0,05, respectively). By contrast, observed reactivity in both, astrocytes and microglia, was drastically decreased in response to DXI treatment independently of diet (GFAP: p < 0,001 APP/PS1 vs APP/PS1 DXI; p < 0.0001 APP/PS1 HFD vs APP/PS1 HFD DXI) and (IBA1: p > 0.0001 APP/PS1 vs APP/PS1 DXI; p < 0.0001 APP/PS1 HFD vs APP/PS1 HFD DXI) (Fig. 6A–D).

### DXI reduces endoplasmic reticulum stress

**Fig. 7:**
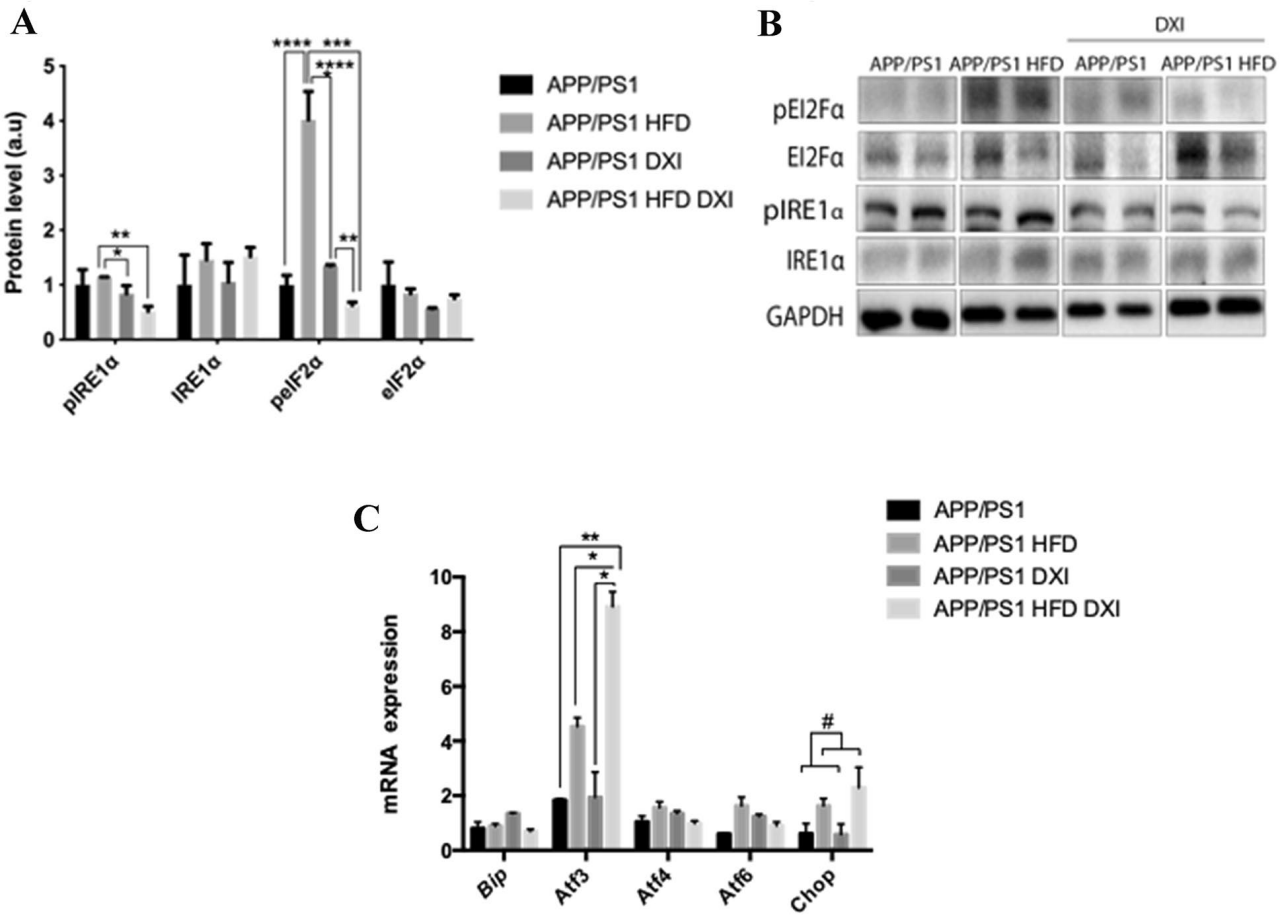
**A**, **B** Immunoblot images and quantification of GAPDH- normalized molecules involved in endoplasmic reticulum stress (pIRE1⍺/IRE1⍺; pEIF2⍺/EIF2⍺) from hippocampal extract (n = 4–6 independent samples per group). **C** mRNA expression profile (n = 4–6 independent samples per group, with 3 technical replicates per sample) of *Bip, Atf3, Atf4, Atf6* and *Chop* from hippocampal extract. Statistical analysis was carried out through two-way ANOVA (*Chop*, diet variable: # denotes p < 0.05) and Tukey´s post-hoc where * denotes p < 0.05; ** denotes p < 0.01; *** denotes p < 0.001 and **** denotes p < 0.0001

Taking into account the involvement of endoplasmic reticulum stress in multiples pathologies among them in inflammatory processes [48] and metabolic disorders [49], we evaluated proteins involved in the main unfolded protein response (UPR) cascades such as inositol-requiring enzyme -1alpha (IRE1α) and eukaryotic initiation factor 2 alpha subunit (eI2Fα). In this line, statistical analysis of pIRE1α confirm a significant effect of treatment (p < 0.01) previously described [49] with interaction between both factors (p < 0.05). Tukey´s post-hoc demonstrated that DXI treatment induced a significant decrease in pIRE1α protein levels in APP/PS1 fed with obesogenic diet compared to their control (p < 0.01). However, in the case of animals fed with conventional chow, although DXI group showed a downward trend, the observed changes were not significant. Regarding to eI2Fα, in line with previous studies [50, 51], two-way ANOVA revealed a significant effect of diet and treatment in peI2Fα protein levels in hippocampus (p < 0.0001) and p < 0.0001) respectively with interaction between both variables ( p < 0.0001). After Tukey´s post-hoc analysis, our results confirmed a significant increase in the phosphorylated levels of this protein due to obesogenic intake in comparison to APP/PS1 mice fed with conventional chow (p < 0.0001), effect which was completely reverted due to DXI treatment (p < 0.0001). No significant effect of either diet nor treatment was observed in IRE1α and eI2Fα protein levels (Fig. 7A, B).

Regarding to mRNA expression, different transcript factors located downstream of IRE1α and eI2Fα in the UPR pathway were analyzed. In this context, no significant effect neither in diet nor treatment variable were observed in activating transcription factor 4 (*Atf4)* and activating transcription factor 6 (*Atf6)* mRNA expression. However, both of them showed interaction between both factors, (p < 0.05) and (p < 0.05) respectively. When analyzing C/EBP homologous protein (*Chop*, statistical analysis demonstrated a significant effect of diet in the expression of this molecule (p < 0.05), confirming the alteration of UPR due to high fat diet intake. Finally, two-way ANOVA of the results of activating transcription factor 3 (*Atf3)* expression showed a significant effect of diet (p < 0.001) and treatment (p < 0.05) with interaction between both variables (p < 0.05). Tukey´s post-hoc multiple comparison indicated a significant increase in APP/PS1 HFD DXI group compared to its control (p < 0.05). No significant difference was observed in binding immunoglobulin protein *(Bip)* expression (Fig. 7C).

### DXI improves insulin signaling pathway APP/PS1 mice hippocampus

**Fig. 8:**
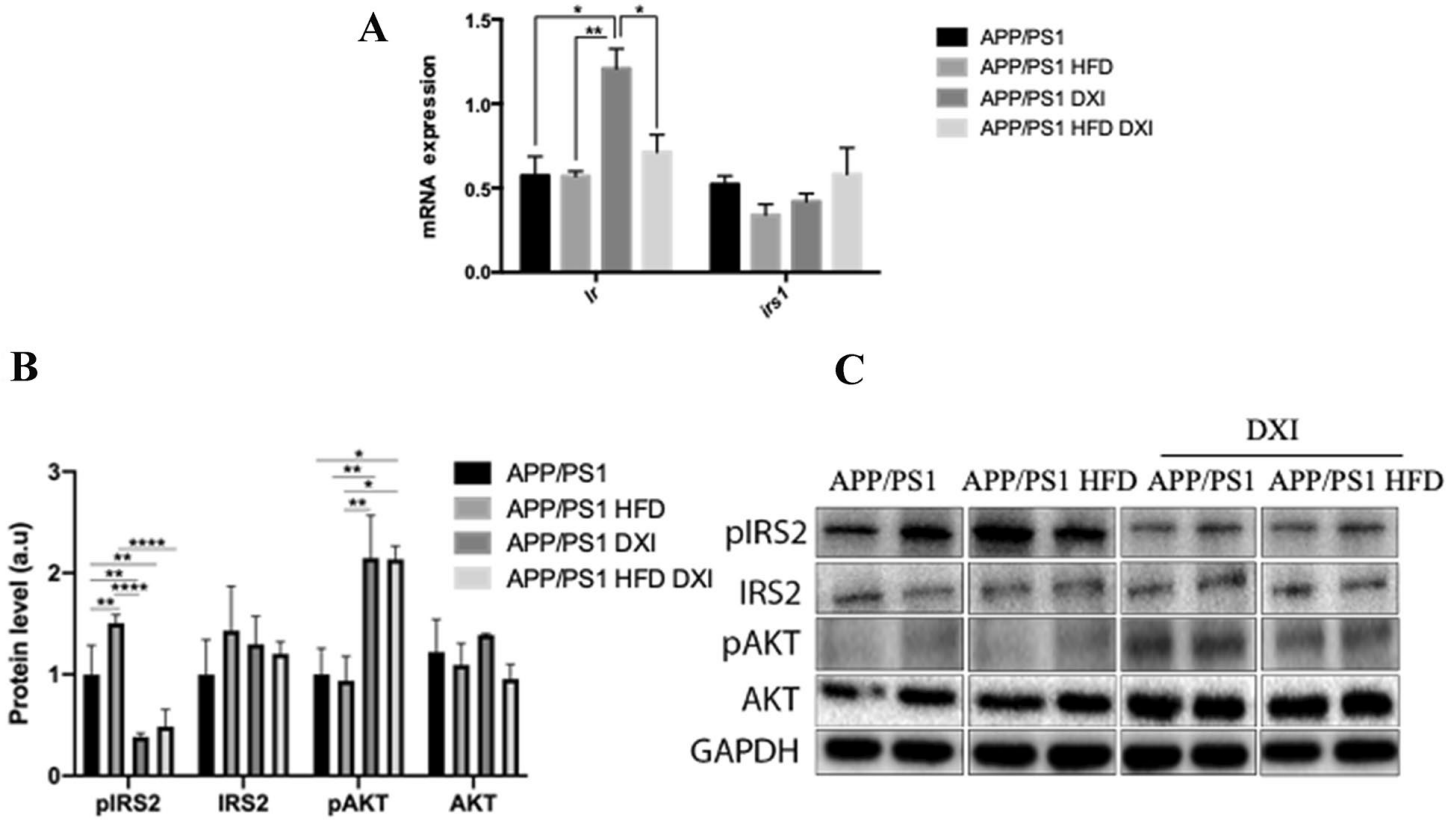
**A** mRNA profile (n = 4–6 independent samples per group, with 3 technical replicates per sample) of *Ir* and *Irs1* from hippocampal extract. **B**, **C** Immunoblot images and quantification of GAPDH- normalized hippocampal molecules involved in insulin signalling pathway (pIRS2/IRS2; pAKT/AKT) (4–6 independent samples per group). Two-way ANOVA was carried out and Tukey’s post-hoc where * denotes p > 0.05; ** denotes p < 0.01; **** denotes p < 0.0001)

In parallel, we evaluated mRNA expression profile of insulin repetor (*Ir)* and insulin receptor substrate 1 (*Irs1)* in the hippocampus due to HFD intake has not been only closely related to alterations in UPR pathway but also to insulin signaling disturbances [49]. Two-way ANOVA analysis revealed a significant effect not only in diet variable (p < 0.05) but also in treatment variable (p < 0.01) with interaction between both factors (p < 0.05) in hippocampal *Ir* mRNA expression. Specifically, Tukey´s post-hoc test indicated that *Ir* transcripts were significantly increased in APP/PS1 animals treated with DXI in comparison to mice without treatment (p < 0.05). Despite this, the effect was blocked by HFD intake (Fig. 8A).

Regarding protein levels, the immunoblotting analysis revealed a significant effect in hippocampal phospho insulin receptor substrate 2 (pIRS2) protein level of diet (p < 0.05) and treatment (p < 0.0001) with interaction between both factors (p < 0.05). Multiple comparison performed indicated a significant increase in pIRS2 in APP/PS1 mice induced by HFD intake (p < 0.01). By contrast, these levels were drastically decreased after DXI treatment in both cases, HFD and conventional chow-fed animals (p < 0.01; p < 0.0001), respectively. In line with these results, our data demonstrated that DXI treatment induced a significant increase of pAKT protein levels independent of the diet that animals had consumed in the 2 way-ANOVA statistical analysis (p < 0.001). AKT results were not significant (Fig. 8B, C).

## Discussion

**Fig. 9:**
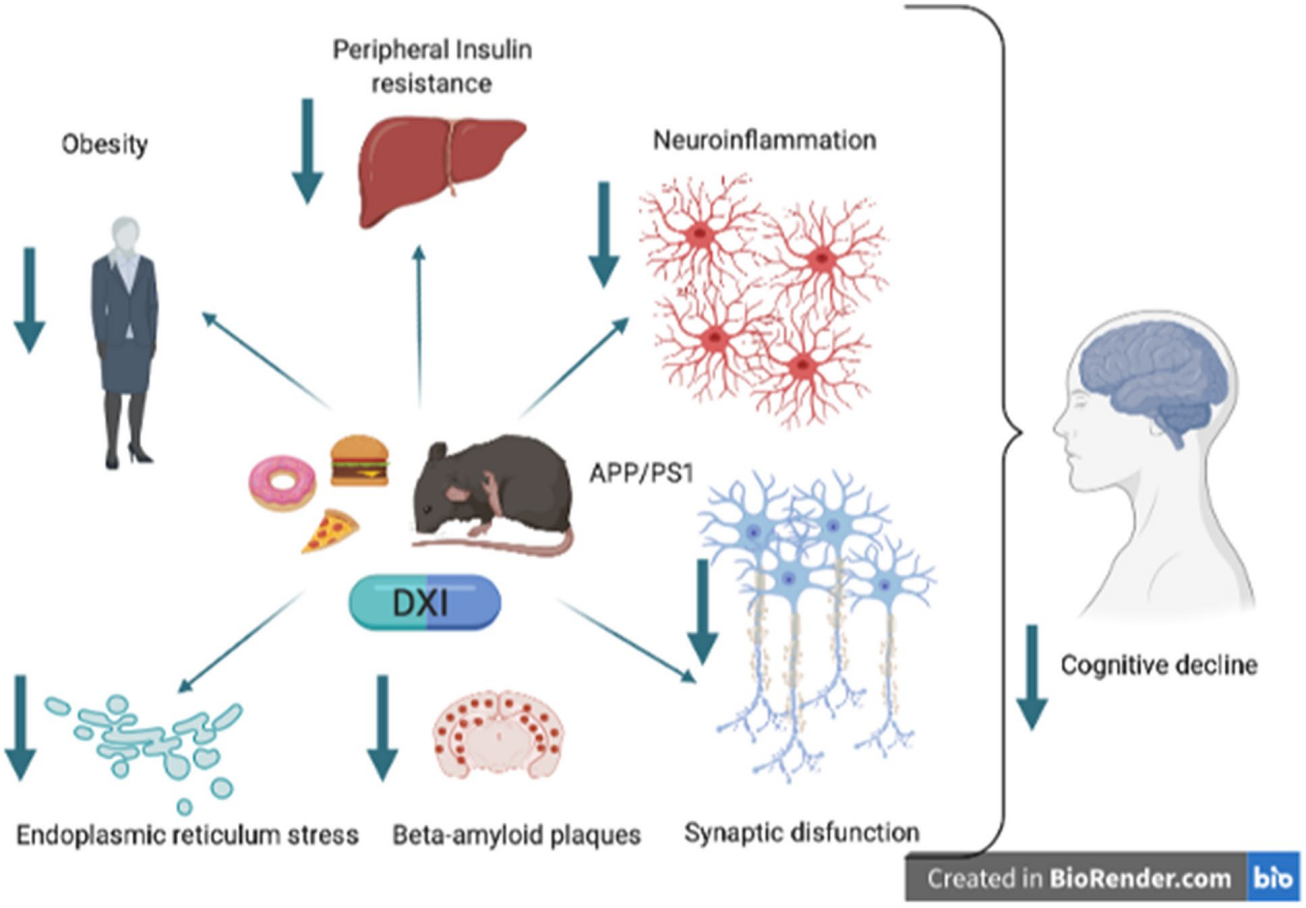
Schematic diagram of the DXI effects. This drug may be an interest compound in the prevention of cognitive loss in AD due to its ability to act on different risk factors. In the present work, where APP / PS1 female mice suffer a metabolic stress associated with a HFD, we demonstrate that DXI improves peripheral parameters associated with insulin resistance. It also improves the cognitive process related to a decrease in the neuroinflammatory process, activation of the non-amyloidogenic pathway, improves the reticulum stress process in addition to parameters related to the synaptic process. All these results suggest that this drug could be used in a combinatorial therapy, for example associated with memantine and acetyl cholinesterase inhibitors for the prevention of late-onset AD

Several epidemiological studies have demonstrated that HFD intake is related to numerous pathologies such as obesity, T2DM and inflammation, all of them strongly involved in the development of cognitive loss in AD [52–55], leading to be considered as a multifactorial disease. Thus, the neuroinflammatory process, associated mainly with microglial activation, could be the bridge between the peripheral initiation of a stress stimuli which will finally end with neuronal loss. In this context, the use of NSAIDs have been proposed as a possible therapeutic strategy to delay or stop the progression of this neurodegenerative disease. Likewise, different epidemiological studies have pointed out the benefits of chronic administration of NSAIDs in terms of reducing the risk for suffering late onset AD [56–59]. Nevertheless, they have not been able to fully achieve their objective, probably due to the late diagnosis of the pathology. Thus, in spite of being well known that inflammation plays a key role in the AD, its contribution to the pathology has not been fully clarified. Our present study evaluates the effect of a chronic administration of DXI in the inflammatory process surrounded AD development, but also in the metabolic alterations such as weight gain, hyperglycemia and insulin resistance induced by HFD intake in APP/PS1 mice. These alterations directly affect the memory process, Aβ deposition, glial reactivity and UPR, as well as in insulin signaling pathway (Fig. 9).

Several studies have revealed that metabolic alterations constitute a high risk factor for the development of late onset AD [60, 61]. These findings have been the key in the approach of alternative point of view in terms of disease etiology and pathophysiology. In this context, several authors have considered AD as a systemic pathology, where brain in not the only responsible of pathological alterations [62–64]. In fact, it has been demonstrated that insulin resistance is a shared hallmark feature of obesity, T2DM and neuropathological processes underlying cognitive aging and dementia [65], together with inflammation. The latter, as it has been previously discussed, plays a key role in these pathological processes [66]. In line with this, there are epidemiological evidence suggesting a correlation between inflammation and insulin resistance state, where it has been described that obesity and the concomitant development of inflammation are the main contributors of insulin resistance. Likewise, studies in human obesity and insulin resistance have revealed a clear relation between chronic administration of pro-inflammatory signaling pathways and decreased insulin sensitivity [67–69]. In accordance with these results, our results confirm that the chronic administration of DXI reduces weight gain, glucose and insulin impaired response and insulin signaling pathway alterations in liver induced by HFD intake in APP/PS1 mice, reverting the metabolic alterations tightly associated to AD development.

Conversely, it is well known that brain gliosis activation plays an essential role in both, the development of all neurodegenerative disorders and metabolic disorders [70]. In fact, several studies have remarked that neuroinflammation is a clear feature of the pathology which is highly enhanced after HFD consumption as it has been demonstrated in our study and also by other research groups [71, 72]. Likewise, it has been described that neuroinflammatory process contributes to synaptic dysfunction and behavioral deficits in early stages of the disease [73, 74]. In this context, our results demonstrate that the chronic administration of DXI drastically reduced the glial reactivity observed in both, APP/PS1 and APP/PS1 HFD mice, leading to the restorage of synaptophysin, DBN1 and behavioral impairments. Regarding to the accumulation of Aβ oligomers, MacPherson and coworkers demonstrated that peripheral administration of tumor necrosis factor alpha (TNFα) inhibitors modified the proinflammatory profile of AD mice, thus rescuing long term-potentiation together with the decrease of Aβ plaques [74]. In line with these previous results, our study confirms that the reduction of the inflammatory response also contributes to the reduction of Aβ burden in the brain. Moreover, altogether confirms the study performed by Ho et al*.* and others, which demonstrated that preclinical AD animal models developed peripheral insulin resistance which coincide with the onset of Aβ accumulation in the brain, reinforcing the hypothesis that connect peripheral alterations with AD hallmarks [75–77].

At the molecular level, our study shows that chronic treatment with DXI increases drastically the active ADAM10 protein levels, which could be related with the Aβ plaques lowering and the cognitive improvement. In fact, ADAM10 has been proposed as a therapeutic target in terms of treating neurodegenerative conditions, including late onset AD, given that its activity has been associated to neuroprotection through Aβ plaques reduction [78]. Likewise, ADAM10 is considered as a relevant enzyme in the synapses since previous studies demonstrated its involvement in the re-modeling of the synaptic spines [79, 80]. Moreover, in accordance with our data, it has been suggested that ADAM10 represents a new way to regulate excitatory neurons in the hippocampus which could explain the improvement observed in our mice after treatment.

It has been described that neuroinflammation also produces endoplasmic reticulum stress, thus leading to UPR disrupted activation [81]. In fact, Pintado and coworkers demonstrated that lipopolysaccharide-induced neuroinflammation produce UPR activation [81], leading to synaptic dysfunction, neuronal plasticity decrease and axonal alteration, all of them contributing to neurodegeneration [82]. In line with this association, chronic activation of the UPR has emerged as a conserved feature among various neurodegenerative in both, animal models and *post-mortem* studies of tissues obtained from patients [83], neurodegenerative disorders which share an accumulation of specific misfolded proteins [84–86]. Specifically, in prion-infected mice, functional studies have demonstrated that sustained phosphorylation of eIF2α dramatically reduces expression of synaptic proteins [87]. Likewise, its suppression has been associated with the relief of AD-related plasticity and memory deficits, demonstrating that the chronic activation of this signaling has adverse effects on synaptic function [88, 89]. These previous data are in accordance with our results, since our data showed not only a significant increase in p-eIF2α protein level in APP/PS1 mice fed with an obesogenic diet in comparison to APP/PS1 mice but also a significant reduction after DXI treatment. Moreover, animals treated with DXI showed a significant decrease in pIRE1α. Furthermore, these data corroborate the studies performed by Duran-Aniotz et al. [90] which demonstrated that the ablation of IRE1α reduced the Aβ deposition and synaptic and cognitive function restorage in AD mice models. Besides, these studies are in line with others which showed the fundamental involvement of this protein in pathological conditions. It is involved not only in neurodegenerative diseases but also in obesity, inflammation and T2DM, among others. Therefore, its inhibition could offer an interesting avenue for AD [91–93]. Going further, *ATF3* is a stress-induced and adaptive-responsive gene located downstream of eIF2α which basal levels are low. However, they can rapidly increase under stress [94, 95]. By contrast, its functions are variable depending on the context, where evidence demonstrates that NSAIDs induce ATF3 expression and activation, conferring neuroprotection through the inflammatory cytokines downregulation [95, 96] and promoting neural outgrowth [97]. In this context, our results supported these previous studies showing a significant increase in *Atf3* mRNA expression in response to the stress caused by the obesogenic diet intake, which is drastically increased due to NSAIDs effect.

Finally, our study demonstrates a significant improvement in the hippocampus insulin signaling pathway, which is in accordance with this reported by Dasgupta and coworkers, who demonstrated that catestin improves insulin sensitivity by attenuating UPR through in silico and in vivo validation [98].

## Conclusions

In summary, the present study confirms for the first time that DXI chronic administration in a mixed model of preclinical obesity associated T2DM and familial AD, improved-related hallmarks associated to both diseases and modifying both central and peripheral targets involved in the development of AD. Our data strongly suggest that DXI could be a promising therapeutic strategy for the modification of the AD course. Due to the complexity of the disease and the multiple risk factors associated to its development where inflammatory process is key, we propose a combinatory therapy of different drugs considering essential the administration of NSAIDs with few side effects such as DXI with the aim to delay AD development (Additional file 1: Fig. S1).

## Supplementary Information

**Additional file 1: Fig. S1.** Speed on the training day (no statistical differences were obtained).

## Data Availability

All data generated or analysed during this study are included in this published article.

## Abbreviations

Aβ: Amyloid beta
AD: Alzheimer´s disease
ADAM10: Disintegrin and metalloproteinase domain -containing protein 10
AKT/ p-AKT: Protein kinase B/ phospho- Protein kinase B
APP/PS1: APPswe/PS1dE9
*Atf3*: Activating factor 3
*Atf4*: Activating factor 4
*Atf6*: Activating factor 6
a.u.: Arbitrary units
*Bip*: Binding immunoglobulin protein
*Chop*: C/EBP homologous protein
DBN1: Drebrin
DI: Discrination index
DXI: Dexibuprofen
EI2Fα/p- EI2Fα: Eukaryotic initiation factor 2 α subunit/ phospho Eukaryotic initiation factor 2 α subunit
FBS: Fetal bovine serum
GAPDH: Glyceraldehyde 3-phosphate dehydrogenase
GD: Gyrus dentatus
GFAP: Glial fibrillary acidic protein
GKS3β/ p-GKS3β: Glycogen synthase kinase 3 β
HFD: High fat diet
IBA1: Ionized calcium binding adaptor molecule 1
IBU: Ibuprofen
IP-GTT: Intraperitoneal-glucose tolerance test
IP-ITT: Intraperitoneal-insulin tolerance test
*Ir*: Insulin receptor
IRE1a/ p-IRE1a: Inositol-requiring enzyme-1a
*Irs1*: Insulin receptor substrate 1
IRS2: Insulin receptor substrate 2
MWM: Morriw water maze
NSAID: Nonsteroidal anti-inflammatory drug
NORT: Novel object recognition test
O/N: Over/night
PB: Phosphate buffer
PBS: Phosphate-buffered saline
PFA: Paraformaldehyde
RT-PCR: Real time PCR
T2DM: Type 2 Diabetes Mellitus
TNFα: Tumor necrosis factor α
UPR: Unfolded protein response
