# Supplementary material for: Dexibuprofen ameliorates peripheral and central risk factors associated with Alzheimer’s disease in metabolically stressed APPswe/PS1dE9 mice
Source: Cell Biosci. 2021 Jul 22;11:141. doi: 10.1186/s13578-021-00646-w (PMC8296685; doi:10.1186/s13578-021-00646-w)
Supplement: Supplementary file 1 — Additional file 1: Fig. S1. Speed on the training day (no statistical differences were obtained). [file 13578_2021_646_MOESM1_ESM.docx]

**Additional file**

**Fig. S1.** Speed on the training day (no statistical differences were obtaine).
